# Silencing PCCA Suppresses CRC Growth and Spread by Modulating EMT and M1 Macrophage Polarization

**DOI:** 10.7150/ijms.102046

**Published:** 2025-01-01

**Authors:** Chuyi Zhang, Zhinan Zheng, Huaiming Wang, Ziwei Qi, Ying Wang, Zhunyi Gao, Yuhui Huang, Sanqing Jin

**Affiliations:** 1 Department of Anaesthesia, The Sixth Affiliated Hospital, Sun Yat-sen University, Guangzhou, China.; 2 Biomedical Innovation Center, The Sixth Affiliated Hospital, Sun Yat-sen University, Guangzhou, China.; 3 Cyrus Tang Medical Institute, Collaborative Innovation Center of Hematology, State Key Laboratory of Radiation Medicine and Prevention, Soochow University, Suzhou, China.; 4 Department of Colorectal Surgery, The Sixth Affiliated Hospital, Sun Yat-sen University, Guangzhou, China.

**Keywords:** Colorectal cancer, Epithelial-mesenchymal transition, Macrophage polarisation, Signal transduction, Propionyl-CoA carboxylase

## Abstract

**Background:** The progression and metastasis of colorectal cancer (CRC) remain major clinical challenges due to a lack of effective therapeutic targets. Our preliminary study identified the upregulation of the propionyl-CoA carboxylase alpha chain (PCCA) gene in CRC, prompting further investigation into its functional roles.

**Methods:** Bioinformatics analysis, colorectal tumor tissues, and CRC cell lines were used to determine PCCA expression. Wound healing, Transwell, and cell counting kit-8 (CCK-8) assays were conducted to evaluate the impacts of PCCA expression on CRC cell migration, invasion, and proliferation. Western blotting was used to assess epithelial-mesenchymal transition (EMT) markers and associated signaling pathways. Mouse models, flow cytometry, and quantitative polymerase chain reaction (PCR) were performed to investigate the influences of PCCA on CRC tumor growth, lung metastasis, and macrophage polarization.

**Results:** PCCA is highly expressed in CRC tumor tissues compared to normal tissues and is associated with a poor prognosis. Knocking down PCCA reduced CRC cell migration, invasion, and proliferation, which were associated with the upregulation of E-cadherin, the downregulation of N-cadherin, Vimentin, and Fibronectin, as well as the inactivation of the extracellular signal-regulated kinase (ERK)/glycogen synthase kinase 3 beta (GSK3β) signaling pathway. Moreover, PCCA knockdown suppressed CRC tumor growth and lung metastasis, accompanied by an increase in M1-macrophage polarization.

**Conclusion:** Knockdown PCCA inhibits the progression and metastasis of CRC, which is associated with EMT reversion, ERK/GSK3β signaling inactivation, and M1-macrophage polarization. These findings suggest that PCCA is a potential target for controlling CRC.

## Introduction

Colorectal cancer (CRC) accounts for approximately 10% of all newly diagnosed cancer cases annually and is the second leading cause of cancer-related mortality worldwide^1^, ^2^. Among individuals diagnosed with CRC, approximately 20% and 40% of patients develop metastatic CRC and experience recurrence following treatments, respectively^3^, ^4^. Advances in medical treatment have enabled curative or long-term disease control of localized CRC^5^. However, the prognosis of metastatic CRC remains dismal, with a median 5-year survival rate of only 18.5% in the United States and 27.7% in Europe^3^, ^6^, ^7^.

Currently, treatment modalities for CRC include surgery, chemotherapy, targeted therapy, and immunotherapy. Accumulating clinical trials have shown that tailoring treatments for CRC according to the molecular and pathological features improves overall survival^3^. However, owing to the heterogeneity of CRC, only a small subset of patients benefits from treatments, especially immunotherapy or targeted therapy^3^, ^8^, ^9^. For example, only around 5% of CRC patients with high microsatellite instability and mismatch repair deficient tumors are sensitive to immunotherapy^9^, ^10^. Therefore, there is an unmet clinical need to identify effective therapeutic targets for CRC.

Our preliminary bioinformatics and clinical sample analyses showed that propionyl-CoA carboxylase alpha chain (PCCA) is highly expressed in CRC and is associated with poor prognosis. PCCA is one of the two subunits of biotin-dependent propionic CoA carboxylase (PCC), a mitochondrial enzyme involved in the catabolism of odd-chain fatty acids, branched-chain amino acids (isoleucine, threonine, methionine, and valine), and other metabolites^11^, ^12^. Odd-carbon fatty acids and branched-chain amino acids undergo conversion to propionyl-CoA by PCC, which is further converted to succinyl-CoA and ultimately generates adenosine triphosphate through β-oxidation^12^-^15^. During tumor progression, the nutrient supply to tumor cells fluctuates constantly. Tumor cells rely on fat metabolism to facilitate rapid proliferation, survival, migration, invasion, and metastasis^16^. Colorectal tumor cells often exhibit dysregulated lipid metabolism^17^. Previous studies reported an association between PCCA upregulation and worse prognosis in breast, gastric, and kidney cancers^18^-^20^. Therefore, we hypothesize that PCCA could be a novel target to control CRC progression.

In this study, *in vitro* and *in vivo* experimental data showed that PCCA upregulation promotes the progression and metastasis of CRC, which is associated with epithelial-mesenchymal transition (EMT), the activation of the extracellular signal-regulated kinase (ERK)/glycogen synthase kinase 3 beta (GSK3β) signaling pathway, and the suppression of M1-macrophage polarization. These findings suggest that PCCA is a potential therapeutic target for CRC.

## Materials and Methods

### Data acquisition

The GSE117606 and CSE11237 datasets were downloaded from the Gene Expression Omnibus (GEO, https://www.ncbi.nlm.nih.gov/geo/). These datasets were analyzed using an Affymetrix HT HG-U133+ PM Array Plate. R software (version 4.1.1) and the limma package (version 3.26.8) were employed to process the data with thresholds set at adjusted *P*-value <0.05 and |log2(Fold Change)| ≥ 0.5. Additionally, the University of California Santa Cruz Xena (UCSC Xena, https://xenabrowser.net/) provides access to gene expression and clinical data for patients with CRC from the COAD and READ datasets in The Cancer Gene Atlas (TCGA) database.

### Bioinformatics analysis

Genes associated with PCCA were analyzed using LinkedOmics (http://www.linkedomics.org/). The results were visualized using volcanic plots and heat maps. Gene ontology enrichment analysis was conducted on the top 50 genes correlated with PCCA, displaying the first 10 results for each analysis.

Tumor purity was evaluated using the “estimate” package in R, which calculated immune, stromal, and ESTIMATE scores, indicating the abundance of tumor-associated components. The percentages of M1 and M2 tumor-associated macrophages (TAMs) were predicted using CIBERSORT (https://cibersortx.stanford.edu/), a tool for deconvolving expression matrices from 22 human immune cell subtypes^21^. The half-maximal inhibitory concentration (IC50) of cisplatinum was calculated using the pRRophetic package in R to reflect the drug response^22^.

### Tumor and lung metastasis models

Female nude mice (6-8 weeks old) were purchased from the Shanghai Laboratory Animal Center and housed under specific pathogen-free conditions at Soochow University. All animal experiments adhered to the institutional guidelines and were approved by the Soochow University Institutional Animal Care and Ethical Committee. For tumor growth studies, 2 × 10^6^ HCT116 cells were subcutaneously injected into the flanks of mice. Each group included 7 mice. Each experiment was repeated 2 times. Tumor dimensions were measured every three days using a Vernier caliper, and the volume was calculated using the following formula: volume = 0.5 × (width^2^ × length). For metastasis studies, HCT116 cells with differential PCCA expression were injected intravenously (i.v.), and the lung metastatic burden was assessed after euthanasia. For the PCCA overexpressed HCT116 cell line and its corresponding control cell line, each group included 9 mice, and the lung tissues were collected for evaluation at 6 weeks after i.v. injection. For the PCCA knockdown HCT116 cell lines and their corresponding control cell line, each group included 10 mice, and the lung tissues were collected for evaluation at 8 weeks after i.v. injection. Each experiment was repeated 2 times.

### Human tissues and cell lines

Six pairs of freshly frozen CRC tissues and adjacent normal tissues were obtained from patients who underwent surgery at the Sixth Affiliated Hospital of Sun Yat-sen University. This study was approved by the Institutional Review Board of the Sixth Affiliated Hospital of Sun Yat-sen University.

CRC cell lines HT29, SW480, DLD1, and RKO, along with normal human colorectal epithelial NCM460 cells, were generously provided by Dr. Ping Lan at the Sixth Affiliated Hospital of Sun Yat-sen University, Guangzhou, China. The HCT116 cells were obtained from the National Collection of Authenticated Cell Cultures. HEK 293T cell lines were obtained from the American Type Culture Collection. HT29, DLD1, NCM460, and 293T cells were cultured in Dulbecco's modified eagle's medium medium (DMEM; Gibco, NY, USA) supplemented with 10% fetal bovine serum (FBS; Hyclone, USA) and 1% penicillin/streptomycin (Gibco, NY, USA). The RKO and SW480 cell lines were cultured in RPMI 1640 medium (Gibco, NY, USA) supplemented with 10% FBS. HCT116 cells were maintained in MycCoy's 5A medium (Gibco, NY, USA) supplemented with 10% FBS. All cell lines were incubated at 37°C in a cell incubator with a 95% air and 5% carbon dioxide mixture (Thermo Scientific, US) and detached using 0.25% trypsin-ethylenediaminetetraacetic acid solution with phenol red (Cat#C0203, Beyotime, China).

### Complementary DNA (cDNA), small interfering RNA (siRNA), and lentivirus infection

The cDNA of PCCA (Tsingke Biotechnology, China) and siRNA plasmids targeting PCCA (Ribobio, China) were transiently transfected into the cells using Lipo8000 (Cat#C0533, Beyotime, China) to regulate PCCA expression. The sequences of the two PCCA siRNAs were as follows: siRNA #1 RNA: GCAAGAAGATGGGCATTAA, and siRNA #2 RNA: GTATCCTGATGGCTTCAAA. The common short hairpin RNA (shRNA) sequence was obtained from Tsingke Biotechnology, utilizing the plko.1-copGFP-puro vector for shRNA and the PDS279-pL-CMV- GFP-ccdb-puro vector for cDNA. Lentiviruses were generated by co-transfecting the aforementioned shRNAs or cDNA with two packaging plasmids (psPAX2 and pMD2.G; Tsingke Biotechnology, China) into 293T cells for 48 hrs using Lipofectamine 3000 (ThermoFisher, USA). Stable transfection cell lines were established by infecting HCT116 cells with the lentivirus at a polybrene concentration of 2 μg/mL. Cells were screened using puromycin at a concentration of 2 mg/mL (Cat# ST551, Beyotime) for subsequent experiments.

### Western blot

Cells were harvested and then lysed by cell lysis buffer (Cat#P0013, Beyotime, China) supplemented with 1% Protein Phosphatase Inhibitor (Cat# FD1002, Fdbio Science, China). Protein concentration was determined using a bicinchoninic acid kit (Cat#P0010, Beyotime, China). Equal amounts of protein were separated into 10% or 12% sodium dodecyl sulfate-polyacrylamide gel electrophoresis gels using a polyacrylamide gel electrophoresis Gel Fast Preparation Kit (Cat#PG112; Epizyme, China). Separated proteins were then transferred to polyvinylidene fluoride membranes (Cat# IPVH00010; Merck Millipore, Billerica, MA, USA). After blocking with Protein-Free Rapid Blocking Buffer (5×) (Cat#PS108, Epizyme, China) diluted in tris-buffered saline with tween-20 buffer for 15 minutes, the membrane was incubated with respective primary antibodies overnight at 4°C. Following three washes with tris-buffered saline for 5 min each, the membrane was incubated with DyLight 800 4X PEG Conjugate IgG (H+L) (1:10000, anti-rabbit, Cat#5151; anti-mouse, Cat#5257, Cell Signaling Technology, USA) for 1 h at room temperature. Finally, protein bands were visualized using a two-color infrared fluorescence imaging system. Antibodies used for western blotting are listed in Table S1. Western blots were done for at least three individual experiments and one representative blot was shown.

### Flow cytometry analysis

Tumor tissues were isolated, and single-cell suspensions were prepared using DMEM medium supplemented with 1500 U/mL collagenase type 1 (Sigma, USA), 1000 U/mL hyaluronidase (Sigma, USA), and 20 U/mL DNase (Sigma, USA) for 45 minutes at 37°C. Before antibody staining, a rat anti-mouse CD16/CD32 monoclonal antibody was added to the single-cell suspensions for 5 min at room temperature. After staining, cells were washed with pre-cooled flow buffer (1% bovine serum albumin, 0.1% sodium azide in PBS), and 7-amino actinomycin D reagent (eBioscience, USA) was added (5 µL per tube) before flow analysis. Flow cytometry data were acquired using a Gallios flow cytometer (Beckman, USA) and analyzed using the Kaluza software (version 1.3). The following fluorescent-labeled or isotype-matched anti-mouse antibodies were used: PE-Cy7-CD11c, APC-CD206, PE-F4/80, APC-Cy7-Gr1, BV421-CD45, and BV510-CD11b (BioLegends).

### Real-time reverse-transcription polymerase chain reaction

Total cellular RNA was isolated using an RNA freeze reagent (Cat#R711-01, Vazyme, China) according to the manufacturer's protocol. The RNA quality and quantity were measured using a NanoDrop spectrophotometer (Cat#ND-2000; Thermo Scientific, MA, USA). RNA was reverse transcribed into complementary DNA using a HiScript III RT SuperMix for quantitative PCR (+gDNA wiper) according to the manufacturer's instructions (Cat#R323-01, Vazyme, China). The Roche LighterCycler 480 was utilized to perform real-time reverse-transcription PCR using a ChamQ SYBR Color quantitative PCR Master Mix (Without ROX) (Cat#Q4421-02, Vazyme, China). Relative gene expression was calculated using the 2-ΔΔCT method and normalized to β-actin. Primers used for quantitative reverse transcription PCR are listed in Table S2.

### Wound healing assay

Positioning marks were strategically placed on the backs of the six-well plates to ensure consistent wound creation for subsequent experiments. The cells (1 × 10^6^) were seeded onto these plates. Upon reaching 90% confluence, wounds of uniform shape were generated across each well using a 200 μL pipette tip. Afterward, the detached cells were eliminated by washing with phosphate-buffered saline (PBS), and a fresh medium was added. Using an inverted microscope, the scratch areas were photographed at 0, 24, and 48 h. ImageJ software version 1.48v (National Institutes of Health, Bethesda, MD, USA) was used to analyze and quantify cancer cell migration. Each experiment was conducted triplicated, and each experiment was repeated 3 times.

### Transwell assay

Transwell migration and invasion assays were conducted using a Boyden chamber with an 8 μm pore size in 24-well plates without or with Matrigel (Cat#356231, Corning, USA), respectively. CRC cells were suspended in 200 μL of the medium at a concentration of 1 × 10^5^ cells and placed in the upper chamber (Cat#725421, Nest, China). The lower chamber was filled with 600 μL of medium supplemented with 10% FBS. After 24 hrs (PCCA overexpression and vector) or 48 hrs (PCCA knockdown and NC), the cells were fixed with 4% paraformaldehyde (PFA) and stained with 0.1% crystal violet solution (Cat#C0121, Beyotime, China, diluted 1:10 in PBS). The cells in three randomly selected fields on the back side of the inserts were counted under a microscope, and the experiment was repeated twice.

### Cell Counting Kit-8 (CCK-8) viability assay

Cell viability was assessed using the CCK-8 assay (Sevenbio, China). Cells were seeded in 96-well plates at a density of 2 × 10^3^ cells per well in 100 μL of medium containing 10% FBS for 24, 48, 96, 108, and 120 hrs. Absorbance was measured at 450 nm after incubating the cells with 10% CCK-8 solution at 37°C for 1 hrs. Cell viability was assessed by calculating the ratio of optical density values of the sample at 24 hrs. Each experiment was conducted with 5 replicates, and each experiment was repeated 3 times.

### Colony formation assay

Cells were seeded into 6-well plates at a density of 500 cells per well and incubated for 7 days. Then the cells were fixed with 4% paraformaldehyde (PFA) and stained with 0.1% crystal violet solution (Cat#C0121, Beyotime, China, diluted 1:10 in PBS). The cells in three randomly selected fields were counted under a microscope, and cell colonies containing more than 50 cells were counted. The experiment was repeated three times.

### Bone marrow-derived macrophage preparation

The bone marrow was flushed out from the murine tibia and femur with DMEM (containing 10% FBS and 1% penicillin/streptomycin) until the bone appeared white. Bone marrow aspirate was filtered through a sterile 70 μm cell strainer and centrifuged at 4°C, 1300 rpm for 8 min. Subsequently, 1 × 10^6^ bone marrow cells were seeded into a six-well plate and cultured in 2 mL of DMEM (containing 10% FBS and 1% penicillin/streptomycin) supplemented with granulocyte-macrophage colony-stimulating factor (15 ng/mL) (315-02-100, PeproTech, USA). The medium was replaced every other day. Numerous adherent cells resembling macrophages were observed on Day 7.

### Macrophage cultured with conditioned media

Cell supernatants were collected after 48 hrs of HCT116 cell culture and centrifuged at 453 g for 10 min. The supernatant was then filtered through a 0.22 μm filter. The conditioned media was normalized to DMEM based on cell counts and supplemented with 10% FBS. The conditioned media were added to the macrophages and cultured for 48 hrs. Total RNA was extracted using an RNA Isolation Kit V2 (Cat#RC112-01, Vazyme, China), followed by real-time reverse transcription PCR analysis. Each experiment was conducted triplicated, and each experiment was repeated twice.

### Statistical analysis

Statistical analyses were performed using SPSS Statistics (version 19.0; SPSS Inc., Chicago, IL, USA), R (v 4.1.1) software, and GraphPad Prism (version 9.0). Mean comparisons between the two groups were conducted using the independent sample *t*-test for normally distributed data or the non-parametric Mann-Whitney U test for non-normally distributed data. One-way analysis of variance was used for multigroup comparisons. Two-way analysis of variance was used for datasets with two categorical variables and quantitative data. Spearman's correlation analysis was used to assess the correlations between two variables. Survival analysis was performed using the Kaplan-Meier test. The Fisher's exact chi-square test was used to analyze categorical data. *P* < 0.05 was considered statistically significant.

## Results

### High expression of PCCA in CRC was associated with poor prognosis

**Figure 1:**
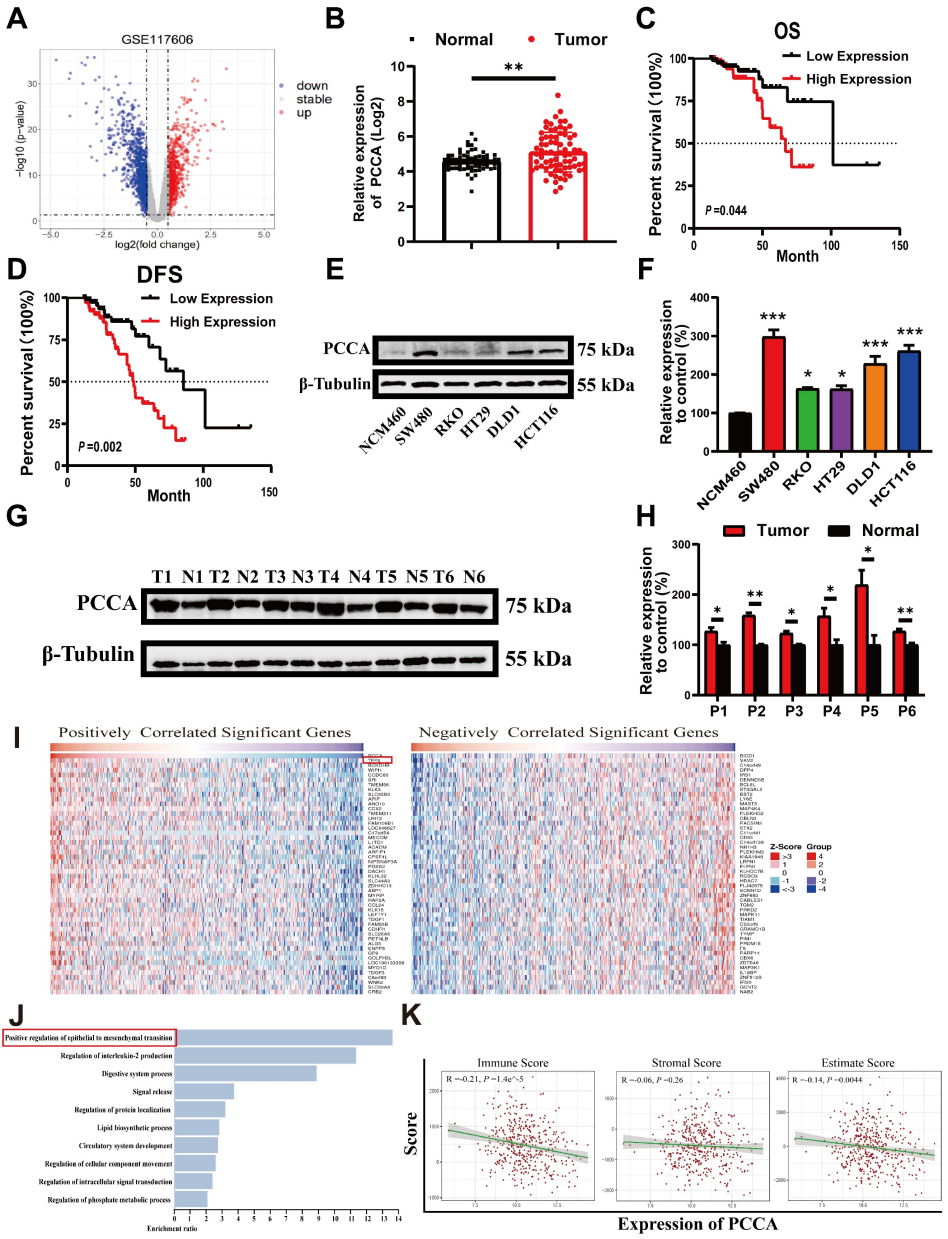
** PCCA was up-regulated in CRC and associated with poor prognosis. (A)** Volcano plots illustrating differentially expressed genes in the GSE117606 dataset. **(B)** Expression levels of PCCA gene in the GEO dataset GSE117606. **(C-D)** OS and DFS of the subgroups of patients with *PCCA* high-expression (n=70) and low-expression (n=84) in the TCGA COAD-READ database. **(E-F)** The representative WB images and the quantification of PCCA expression in CRC cell lines (SW480, RKO, HT29, HCT116, DLD1) and normal colorectal epithelial cells (NCM460)**. (G-H)** The representative WB images and the quantification of PCCA expression in 6 pairs of CRC tumor tissues (T) and adjacent normal tissues (N) from patients.** (I)** The heatmap displays the top 50 positively and negatively correlated genes with PCCA in CRC, and “TFF3” is highlighted in red boxes. **(J)** Gene Ontology (GO) enrichment analysis of *PCCA*-related genes, with “Positive regulation of epithelial to mesenchymal transition” prominently featured. **(K)** The correlation between *PCCA* levels and ESTIMATE scores, including Immune, Stromal, and overall Estimate Scores. The data are presented as means ± SEM (*P < 0.05; ** *P* < 0.01; ***P < 0.001). OS: overall survival, DFS: disease-free survival.

Analysis of GSE117606 data from the GEO database revealed significantly higher levels of PCCA in CRC tumor tissues compared to adjacent normal tissues (Figure 1A, B). Data from the TCGA database also showed that higher PCCA expression was associated with poorer overall survival (OS) and disease-free survival (DFS) (Figure 1C, D). Higher PCCA expression was further validated in CRC cell lines (Figure 1E, F) and CRC tissues from patients (Figure 1G, H).

Next, we explored the relationship between the PCCA gene and malignant phenotypes of CRC. Using LinkedOics, we identified the top 50 genes positively and negatively correlated with PCCA (Figure 1I). Notably, "TFF3" emerged as a top-related gene known to be associated with EMT. Gene ontology enrichment analysis indicated that EMT was the predominant biological process (Figure 1J). Additionally, we used ESTIMATE Score analysis to examine the relationship between PCCA and tumor purity (Figure 1K). These results suggested a potential link between PCCA and reduced immune cell infiltration in CRC. Collectively, these findings indicate the protumoral effects of PCCA in CRC.

### Knockdown of PCCA reduced the migration, invasion, and proliferation of CRC cells

**Figure 2:**
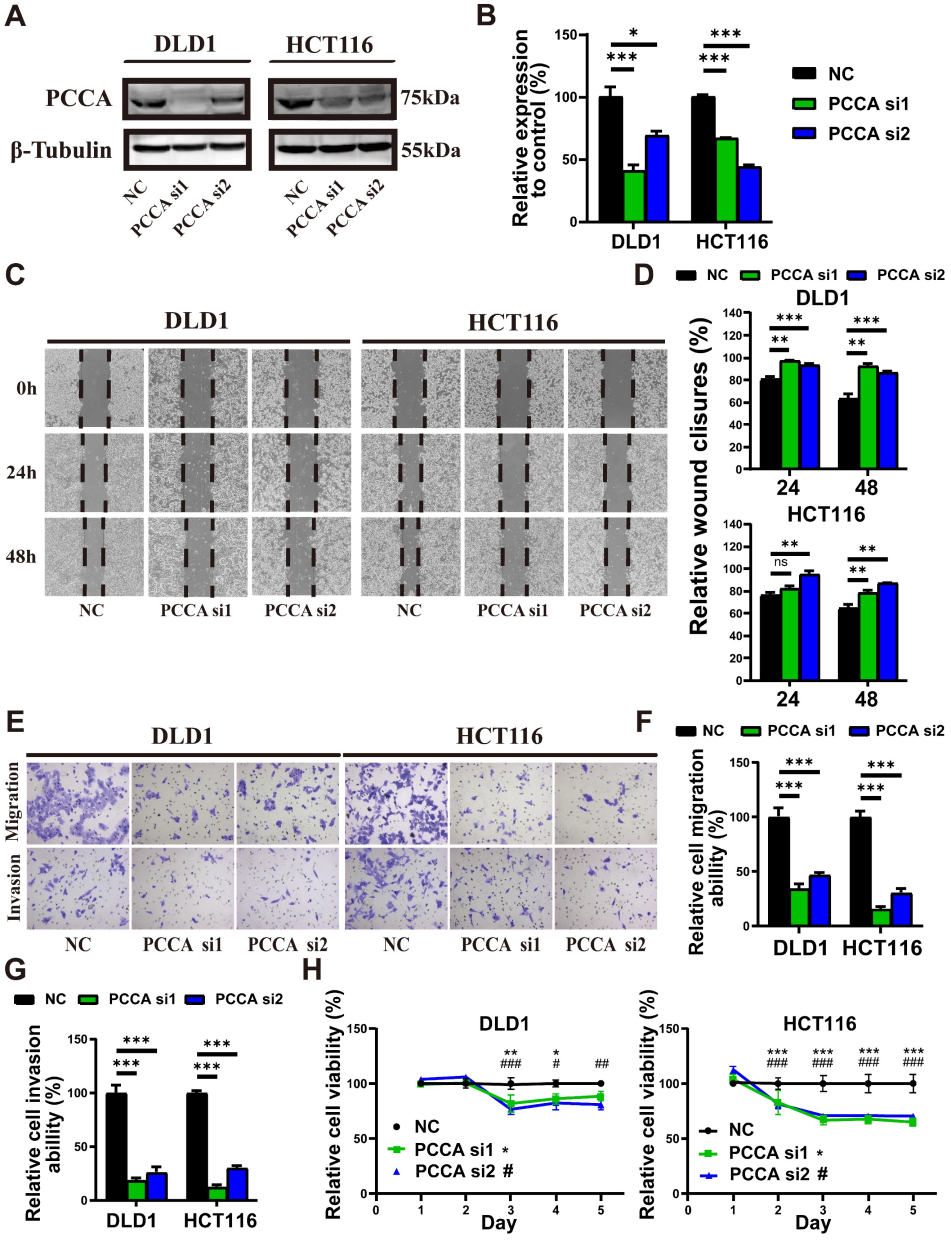
** Knockdown PCCA inhibited the migration, invasion, and proliferation of CRC cells. (A-B)** The representative WB images and the quantification of PCCA expression in DLD1 and HCT116 cells transfected with PCCA siRNA and NC RNA. **(C-D)** The representative images at indicated time points (0 hr, 24 hrs, 48 hrs) and the analysis of wound healing assays conducted on DLD1 and HCT116 cells transfected with *PCCA* siRNA and NC RNA. **(E-G)** The representative images and the analysis of transwell migration and invasion assays performed on DLD1 and HCT116 cells transfected with *PCCA* siRNA and NC RNA.** (H)** The proliferation of DLD1 and HCT116 cells transfected with *PCCA* siRNA and NC RNA was measured by CCK8 assays. Data are from one experiment representative of two **(E-G)** or three **(A-D, H)** independent experiments with similar results. PCCA si1, CRC cells transfected with *PCCA* siRNA-1; PCCA si2, CRC cells transfected with *PCCA* siRNA-2; NC, CRC cells transfected with NC RNA. The data are presented as Means ± SEM (*P < 0.05; ** *P* < 0.01; ***P < 0.001).

HCT116 and DLD1 cell lines were selected for further experiments due to their differential metastatic potential and moderate levels of PCCA expression, which were suitable for manipulation. WB confirmed the downregulation and overexpression of the PCCA protein in both cell lines (Figure 2A, B and Figure S1A, B). Wound-healing (Figure 2C, D and Figure S1C, D) and Transwell assays (Figure 2E-G and Figure S1E-G) demonstrated that PCCA knockdown reduced, while overexpression increased the migratory and invasive capabilities of both cell lines. Knockdown PCCA inhibited HCT116 cell proliferation, with a less pronounced effect on DLD1 cells (Figure 2H and Figure S1H). These results suggest that PCCA facilitates CRC cell migration, invasion, and proliferation.

### Knockdown of PCCA inhibited the EMT and inactivated the ERK1/2 and GSK3β signaling in CRC cells

**Figure 3:**
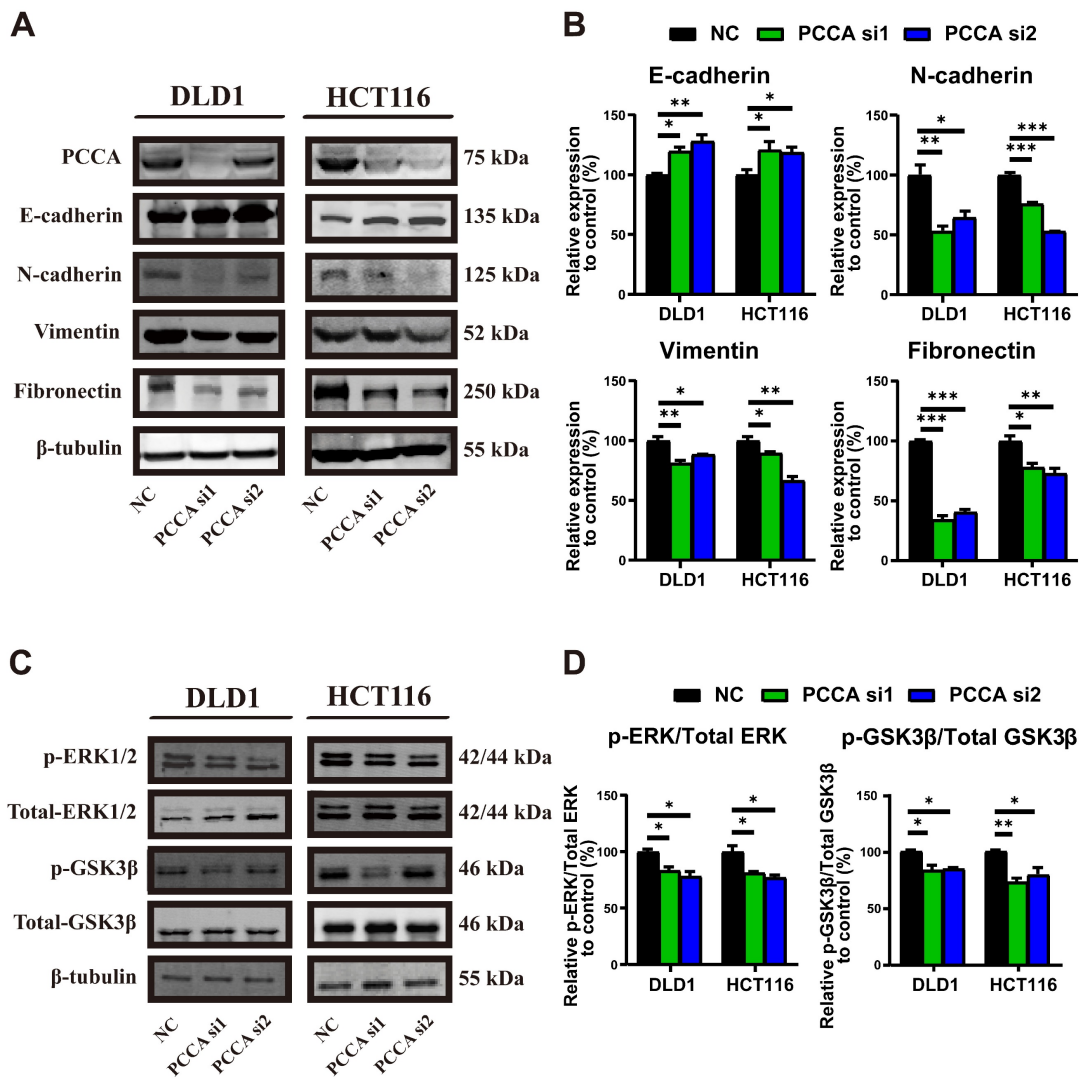
** Knockdown PCCA suppressed the EMT and inactivated the ERK1/2 and GSK3β signaling in CRC cells. (A-B)** The representative WB images and the quantification of the expression of epithelioid markers (E-cadherin) and mesenchymal markers (N-cadherin, Vimentin, and Fibronectin) in DLD1 and HCT116 cells transfected with *PCCA* siRNA and NC RNA. **(C-D)** The representative WB images and the quantification of the phosphorylation of ERK1/2 and GSK3β signaling in DLD1 and HCT116 cells transfected with PCCA siRNA and NC RNA. Data are from one experiment representative of three independent experiments with similar results. PCCA si1, CRC cells transfected with *PCCA* siRNA-1; PCCA si2, CRC cells transfected with *PCCA* siRNA-2; NC, CRC cells transfected with NC RNA. The data are presented as Means ± SEM (*P < 0.05; ** *P* < 0.01; ***P < 0.001).

We then examined epithelial-mesenchymal markers (Figure 3A, B and Figure S2A, B). Knockdown PCCA markedly induced epithelial markers and suppressed mesenchymal markers in CRC cells, whereas overexpression PCCA had the opposite effect. Given the critical roles of ERK1/2 and GSK3β signalings in promoting tumor malignancy^23^-^25^. We also analyzed their phosphorylation. Knockdown PCCA was reduced, while its overexpression elevated the phosphorylation levels of ERK1/2 and GSK3β proteins (Figure 3C, D and Figure S2C, D). Together, these findings suggest that PCCA facilitates EMT and induces ERK1/2 and GSK3β phosphorylation.

### Knockdown of PCCA inhibited tumor growth and lung metastasis of CRC

**Figure 4:**
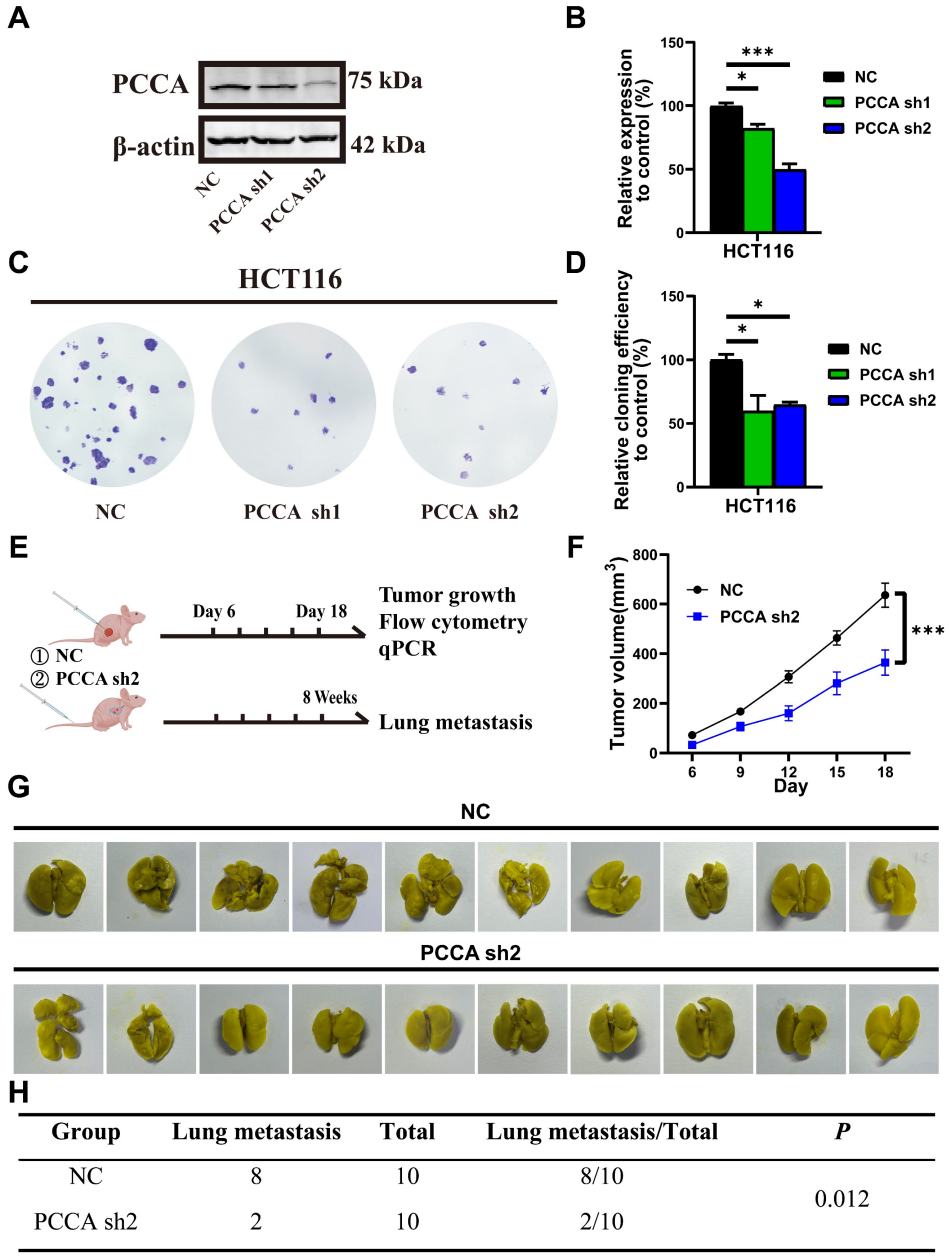
** Knockdown PCCA suppressed CRC tumor growth and lung metastasis. (A-B)** The representative WB images and the quantification of PCCA expression in HCT116-PCCA sh2 or NC. **(C-D)** The representative images and the quantification of the colony formation assay were performed in HCT116-PCCA sh2 or NC. **(E)** The diagram of the subcutaneous tumor and lung metastasis models. **(F)** The tumor growth curves of HCT116-PCCA sh2 or NC in nude mice (n=7). **(G-H)** The images and results of lung metastasis after 8 weeks of tumor cell tail vein injection (n=10). Data are from one experiment representative of two (**E-H**) or three (**A-D**) independent experiments with similar results. PCCA sh2, stable transformation strain of HCT116 with *PCCA* sh2RNA; NC, stable transformation strain of HCT116 with NC RNA. The data are presented as Means ± SEM (*P < 0.05; ** *P* < 0.01; ***P < 0.001).

These results indicate that PCCA had a more potent impact on HCT116 cells compared to that of DLD1 cells. Therefore, we created stable PCCA knockdown and overexpression in HCT116 cells using lentivirus (Figure 4A, B and Figure S3A, B). PCCA overexpression enhanced, while PCCA knockdown was reduced, the clonogenicity of HCT116 cells (Figure 4C, D and Figure S3C, D). Using the stable PCCA sh2 knockdown cell line, which exhibited the most effective knockdown, we established subcutaneous and tail vein lung metastasis models in nude mice (Figure 4E and Figure S3E). PCCA knockdown slowed tumor growth (Figure 4F) and reduced lung metastasis (Figure 4G, H), whereas PCCA overexpression promoted tumor growth (Figure S3F) and lung metastasis (Figure S3G, H). The *in vivo* data demonstrate the pro-tumoral effects of PCCA in CRC.

### Knockdown PCCA facilitated TAM M1-polarization in CRC

**Figure 5:**
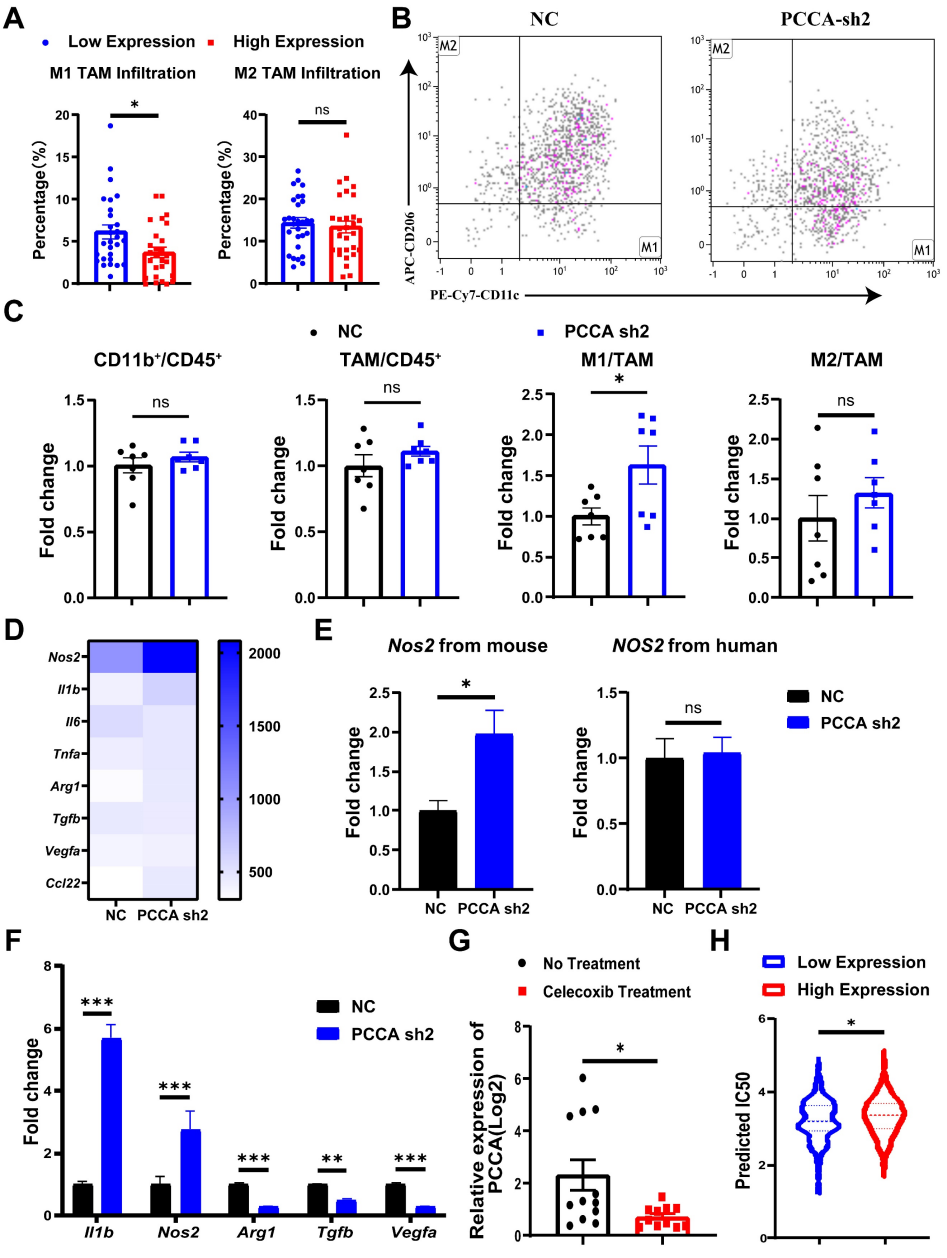
** Knockdown PCCA increased the polarization of TAMs to an M1-like phenotype. (A)** CIBERSORT was utilized to predict the percentages of M1- and M2-like TAMs in the subgroups of metastatic CRC patients with high (29 cases) or low (29 cases) levels of *PCCA* in the TCGA database. **(B)** The representative flow cytometry dot plots depicting the polarization of TAMs in the tumor tissues of HCT116-PCCA sh2 or NC (n=7). **(C)** The statistical analysis of the proportions of myeloid cells (CD11b^+^) and TAMs in leukocytes (CD45^+^), as well as the fold changes of M1-like (CD11c^+^CD206^-^) and M2-like (CD11c^-^CD206^+^) TAMs in the tumor tissues of HCT116-PCCA sh2 or NC (n=7). (**D**) Heatmap showing the transcription levels of M1 and M2 marker genes in the tumor tissue of HCT116-PCCA sh2 or NC. **(E)** The transcription levels of *Nos2* (from mice) and *NOS2* (from humans) in the tumor tissues of HCT116-PCCA sh2 or NC (n=7).** (F)** The transcription levels of M1 and M2 TAM marker genes in bone marrow-derived macrophages co-cultured with the condition media from HCT116-PCCA sh2 or NC tumor cell cultures. The experiment was conducted in triplicates. **(G)** The levels of the PCCA gene in the GSE11237 dataset. **(H)** The violin plot illustrated the estimated IC50 for cis-platinum in the subgroups of CRC patients with high (190 cases) or low (191 cases) levels of PCCA in the TCGA COAD-READ dataset. PCCA sh2, stable transformation strain of HCT116 with *PCCA* sh2RNA; NC, stable transformation strain of HCT116 with NC RNA. The data are presented as Means ± SEM (*P < 0.05; ** *P* < 0.01; ***P < 0.001).

In nude mice with CRC tumors, the tumors in the control group typically exhibited central necrosis, a feature not observed in tumors with PCCA overexpression. This difference might be linked to inflammation. We analyzed the myeloid populations within the tumor microenvironment (TME). The proportions of neutrophils, monocytes, and TAMs were comparable between the two tumor groups. Given that TAMs can exhibit either pro-inflammatory M1-like or anti-inflammatory M2-like phenotypes^26^, ^27^, CIBERSORT analysis of TCGA data indicated lower M1 TAM proportions in tumors with higher levels of PCCA among 58 metastatic CRC cases (Figure 5A). The flow cytometry data showed that PCCA knockdown promoted TAM M1-polarization, while PCCA overexpression exhibited an opposite effect (Figure 5B, C, Figure S4A, B).

Additionally, quantitative PCR analyzed the genes related to TAM polarization (M1 markers: *Nos2*,* Il1b*,* Il6,* and *Tnfa*; M2 markers: *Arg1*,* Tgfb*,* Vegfa,* and* Ccl22*) and the data showed that the transcription levels of *Nos2* were significantly increased in PCCA knockdown tumor tissues (Figure 5D, E), which was reversed in the tissues with PCCA overexpression (Figure S4C, D). Co-culture experiments further showed that alterations in *PCCA* expression influenced TAM polarization (Figure 5F and Figure S4E). Taken together, the data suggest that PCCA suppresses TAM M1-polarization in CRC tumors.

### PCCA may serve as a potential target to improve the therapeutic efficacy of CRC

To further explore the roles of PCCA in CRC, the analysis of the GSE11237 dataset revealed a significant decrease in PCCA gene expression in CRC tumor tissues after celecoxib (a non-steroidal anti-inflammatory drug) administration (Figure 5G). Non-steroidal anti-inflammatory drugs primarily target cyclooxygenase-2 with broad anti-tumor activities^28^, potentially involving PCCA. In addition, using the pRRophetic package, we found a correlation between cisplatin IC50 values and PCCA levels in CRC samples from the TCGA dataset. The results indicated that samples with higher PCCA levels had significantly elevated cisplatin IC50 values compared to those with lower PCCA expression. Considering the crucial role of platinum drugs in CRC chemotherapy^29^, our findings suggest that the levels of PCCA might affect the sensitivity of CRC to cisplatin therapy (Figure 5H).

## Discussion

The significance of metabolic reprogramming involving branched-chain amino acids, lipids, and cholesterol has been highlighted in various tumors^30^-^32^. PCCA, an enzyme involved in the catabolism of odd-chain fatty acids and multiple-branched amino acids, has been implicated in tumor progression^18^-^20^. Our findings reveal that PCCA is upregulated in CRC and correlates with poor prognosis. PCCA may promote CRC progression and metastasis through EMT, the activation of ERK1/2 and GSK3β signaling pathways, and the suppression of TAM M1-polarization. Thus, PCCA could serve as a promising target for CRC treatment.

Previous reports have suggested pro-tumoral effects of lipid-related processes. Wang *et al.* showed the activation of a pathway related to fatty acid oxidation in colorectal tumor cells, thereby facilitating colorectal tumor metastasis^33^. Additionally, circulating branched-chain amino acids are increased in both human and mouse tumor models^34^. Moreover, branched-chain keto dehydrogenase kinase, an enzyme involved in the degradation and utilization of branched-chain amino acids, is highly expressed in colorectal tumors and promotes CRC growth and metastasis in mice^35^, ^36^. Highly proliferative tumor cells exhibit a strong affinity for these nutrients^37^, ^38^. Given that PCCA regulates the metabolism of fatty acids and branched-chain amino acids, these observations may support the pro-tumoral effects of PCCA in CRC.

Our study revealed that PCCA knockdown inhibited the migration and invasion in both DLD1 and HCT116 cells, even though HCT116 is a highly metastatic cell line and DLD1 is less metastatic. However, PCCA knockdown suppressed the proliferation of HCT116 cells but did not significantly impact DLD1 cells. The differential responses indicate that cell migration and invasion could more heavily rely on the oxidative metabolism of fatty acids. Indeed, some reports indicate that colorectal tumor cells with an increased propensity to enter the circulation and cause metastasis rely more heavily on fatty acid oxidation compared to non-free tumor cells^39^. Furthermore, studies have indicated that both metastatic melanoma and ovarian cancer exhibit shifts in energy metabolism toward fatty acid oxidation^34^, ^40^. This shift may occur because conventional glucose metabolism is insufficient to meet the high nutritional demands of highly metastatic tumor cells, prompting them to rely on fatty acids for survival. Consequently, the impacts of PCCA were more pronounced on migration and invasion than on proliferation.

As EMT has been linked to the invasion and metastasis of various cancers^41^-^43^, we also analyzed EMT markers and related signaling pathway activation under varying PCCA expression levels. EMT initiates the first step in tumor metastasis through coordinated signal transduction pathways^44^-^47^. ERK serves as a crucial activator of EMT, triggering this process via diverse mechanisms^48^-^50^, including direct activation of zinc finger E-box binding homeobox 1^51^. Additionally, GSK-3β, a constitutively active kinase, participates in various biological events and collaborates with ERK to regulate multiple cellular processes, notably EMT^52^-^55^. Bioinformatics analysis indicated a strong correlation between PCCA and EMT. Our data study supports that PCCA significantly influences colon cancer progression and metastasis through the modulation of EMT and the ERK/GSK-3β signaling pathway.

Furthermore, we investigated the association between PCCA expression and tumor immunity, particularly focusing on TAMs. These cells are abundant in the TME and are known to be involved in tumor progression, metastasis, and treatment resistance^56^. TAMs undergo metabolic reprogramming and polarize toward M1- or M2-like phenotypes^57^. M1 TAMs exert anti-tumor effects by recognizing and eliminating cancer cells through phagocytosis and cytotoxicity^58^, primarily relying on glucose conversion, lactate production, and reactive oxygen species generation^59^. While our study using nude mice to investigate the tumor immune microenvironment has inherent limitations, these mice could be used to examine changes in tumor-associated myeloid cells, including TAMs^60^-^62^. Our findings reveal that PCCA knockdown led to an increased proportion of M1 TAMs and such effects were reversed by PCCA overexpression.

Notably, murine *Nos2* expression in tumor tissues was altered by PCCA, while human NOS2 expression remained unaffected. NOS2, a key marker of M1 TAMs^63^, synthesizes nitric oxide, which can play dual roles in tumor progression and inhibition depending on the context^64^, ^65^. Our results suggest that altered macrophage polarization contributes to increased *Nos2* expression. We speculated that tumor cells release substances into the peripheral environment, affecting TAM polarization, a conjecture supported by co-culture experiments. Although *in vitro* and *in vivo* results diverged slightly, likely because of other components in the TME, our experiments underscored the impact of *PCCA* expression on TAM polarization, particularly M1 TAMs.

In summary, our data showed that PCCA is upregulated in CRC and is correlated with tumor progression. PCCA increases the malignance of CRC cells, which is associated with EMT and the activation of ERK1/2 and GSK3β signaling pathways. Additionally, PCCA inhibits the polarization of TAMs towards an M1-like phenotype. Notably, PCCA knockdown reverses the above effects. Our findings suggest that PCCA could serve as a potential therapeutic target for CRC.

## Supplementary Material

Supplementary figures and tables.

## Abbreviations

CRC: colorectal cancer
PCCA: propionyl-CoA carboxylase alpha chain
PCC: propionic CoA carboxylase
EMT: epithelial-mesenchymal transition
ERK: extracellular signal-regulated kinase
GSK3β: glycogen synthase kinase 3 beta
GEO: gene expression omnibus
TCGA: the cancer gene atlas
TAM: tumor-associated macrophage
IC50: the half maximal inhibitory concentration
OS: overall survival
DFS: disease-free survival
TME: tumor microenvironment
