# Supplementary material for: Silencing PCCA Suppresses CRC Growth and Spread by Modulating EMT and M1 Macrophage Polarization
Source: Int J Med Sci. 2025 Jan 1;22(1):87–100. doi: 10.7150/ijms.102046 (PMC11659836; doi:10.7150/ijms.102046)

Supplementary Table S1. List of antibodies.

Antigens	Species antibodies raised in	Dilution (WB)	Supplier
PCCA	Rabbit, Polyclonal	1:3000	Proteintech, China, Cat. #21988-1-AP
CDH1/E-Cadherin	Rabbit, polyclonal	1:3000	Boster, China, Cat. # PB9561
CDH2/N-Cadherin	Rabbit, monoclonal	1:1000	Boster, China, Cat. # BM3921
Vimentin	Mouse, monoclonal	1:2000	Boster, China, Cat. # BM0135
Fibronectin	Rabbit, monoclonal	1:3000	Cell Signaling Technology, USA, Cat. #26836
p44/42 MAPK (Erk1/2)	Rabbit, monoclonal	1:3000	Cell Signaling Technology, USA, Cat. #4695
Phospho-p44/42 MAPK (Erk1/2)	Rabbit, monoclonal	1:3000	Cell Signaling Technology, USA, Cat. # 4370
GSK-3β	Rabbit, monoclonal	1:3000	Cell Signaling Technology, USA, Cat. #12456
Phospho-GSK-3β	Rabbit, monoclonal	1:3000	Cell Signaling Technology, USA, Cat. # 5558
β-actin	Mouse, monoclonal	1:2000	Boster, China, Cat. # BM0627
β-tubulin	Mouse, monoclonal	1:3000	Beyotime, China, Cat. #AF2835

Supplementary Table S2. List of primers for quantitative PCR.

Gene	Primer	Sequence (5'-3')
m*Nos2*	Forward	CCACCTCTATCAGGAAGAAA
	Reverse	CTGCACCGAAGATATCTTCA
m*Il1b*	Forward	TGCCACCTTTTGACAGTGAT
	Reverse	TGTCCTCATCCTGGAAGGTC
m*Il6*	Forward	TGATGGATGCTACCAAACTGGA
	Reverse	GGAGAGCATTGGAAATTGGGG
m*Tnfa*	Forward	CCGATGGGTTGTACCTTGTC
	Reverse	CGGACTCCGCAAAGTCTAAG
m*Arg1*	Forward	CAACCAGCTCTGGGAATCTG
	Reverse	AATCGGCCTTTTCTTCCTTC
m*Tgfb*	Forward	ACGTCAGACATTCGGGAAGC
	Reverse	TGTATTCCGTCTCCTTGGTTCAG
m*Vegfa*	Forward	CGGGCCTCGGTTCCAG
	Reverse	TGAACTTGATCACTTCATGGGACT
m*Ccl22*	Forward	GTCCTTCTTGCTGTGGCAAT
	Reverse	ACGGTTATCAAAACAACGCC
m*B-actin*	Forward	GCCACTGTCGAGTCGCGT
	Reverse	GGAGTCCTTCTGACCCATTCC
hNOS2	Forward	CAAGTTCCATCTTTCACCCAC
	Reverse	CCCAGCCTCAAGTCTTATTTC
hGAPDH	Forward	GGAGCGAGATCCCTCCAAAAT
	Reverse	GGCTGTTGTCATACTTCTCATGG

Supplementary Figure S1. Overexpression PCCA promoted the migration, invasion, and proliferation of CRC cells

(A-B) The representative WB images and the quantification of PCCA expression in DLD1 and HCT116 cells transfected with PCCA cDNA and vector plasmid. **(C-D)** The representative images at indicated time points (0 hr, 24 hrs, 48 hrs) and the analysis of wound healing assays conducted on DLD1 and HCT116 cells transfected with *PCCA* cDNA and vector plasmid. **(E-F)** The representative images and the analysis of transwell migration and invasion assays were performed on DLD1 and HCT116 cells transfected with PCCA cDNA and vector plasmid. **(G)** The proliferation of DLD1 and HCT116 cells transfected with PCCA cDNA and vector plasmid was measured by CCK8 assays. Data are from one experiment representative of two **(E-G)** or three **(A-D, H)** independent experiments with similar results. PCCA, CRC cells transfected with *PCCA* cDNA plasmid; Vector, CRC cells transfected with vector plasmid. The data are presented as Means ± SEM (*$P < 0.05$; ** $P < 0.01$; ***$P < 0.001$).

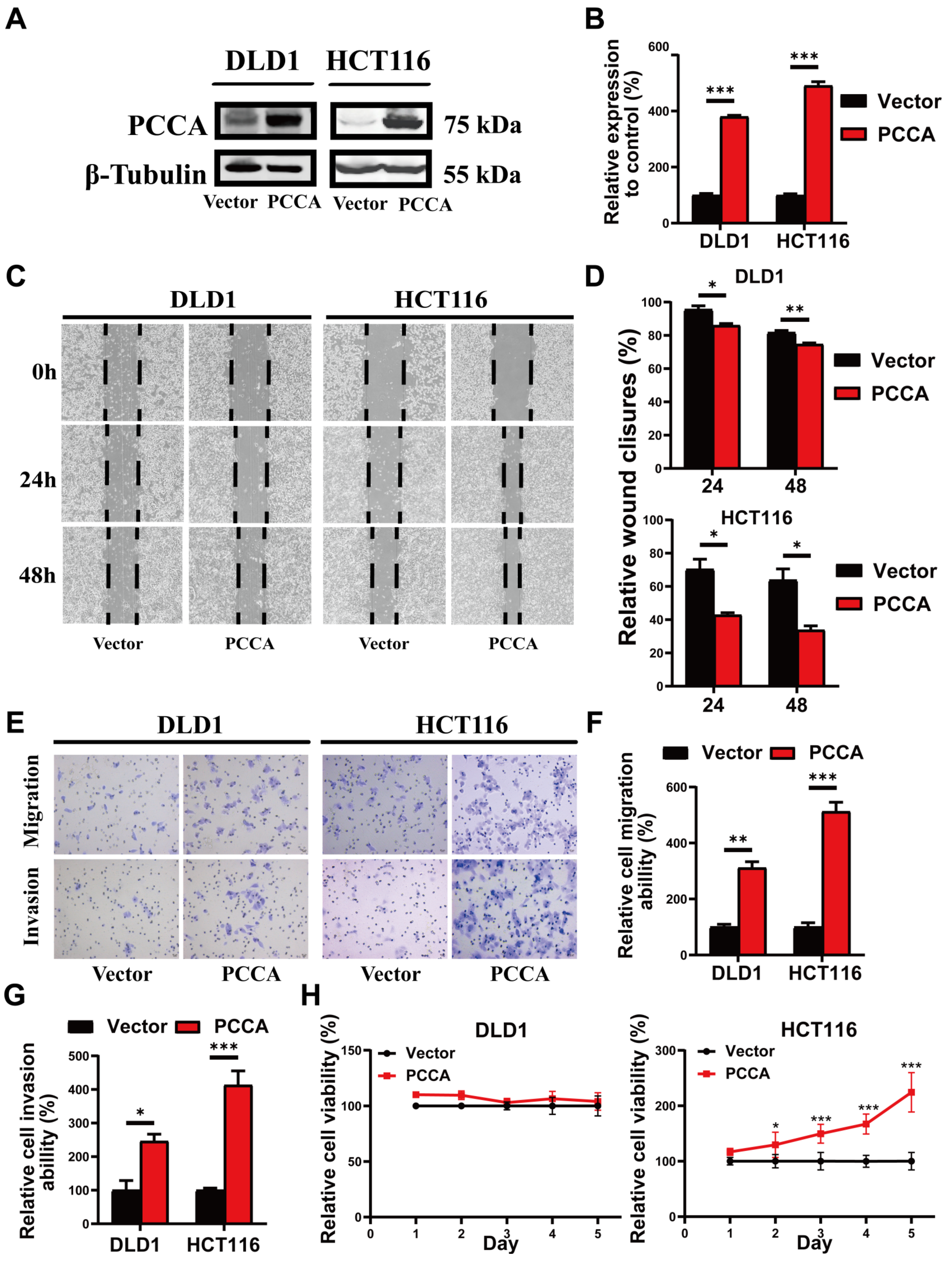

Supplementary Figure S2. Overexpression PCCA promoted the EMT and activated the ERK1/2 and GSK3β signaling in CRC cells

(A-B) The representative WB images and the quantification of the expression of epithelioid markers (E-cadherin) and mesenchymal markers (N-cadherin, Vimentin, and Fibronectin) in DLD1 and HCT116 cells transfected with *PCCA* cDNA and vector plasmid. **(C-D)** The representative WB images and the quantification of the phosphorylation of ERK1/2 and GSK3β in DLD1 and HCT116 cells transfected with *PCCA* cDNA and vector plasmid. Data are from one experiment representative of three independent experiments with similar results. PCCA, CRC cells transfected with *PCCA* cDNA plasmid; Vector, CRC cells transfected with vector plasmid. The data are presented as Means ± SEM (**P* < 0.05; ** *P* < 0.01; ****P* < 0.001).

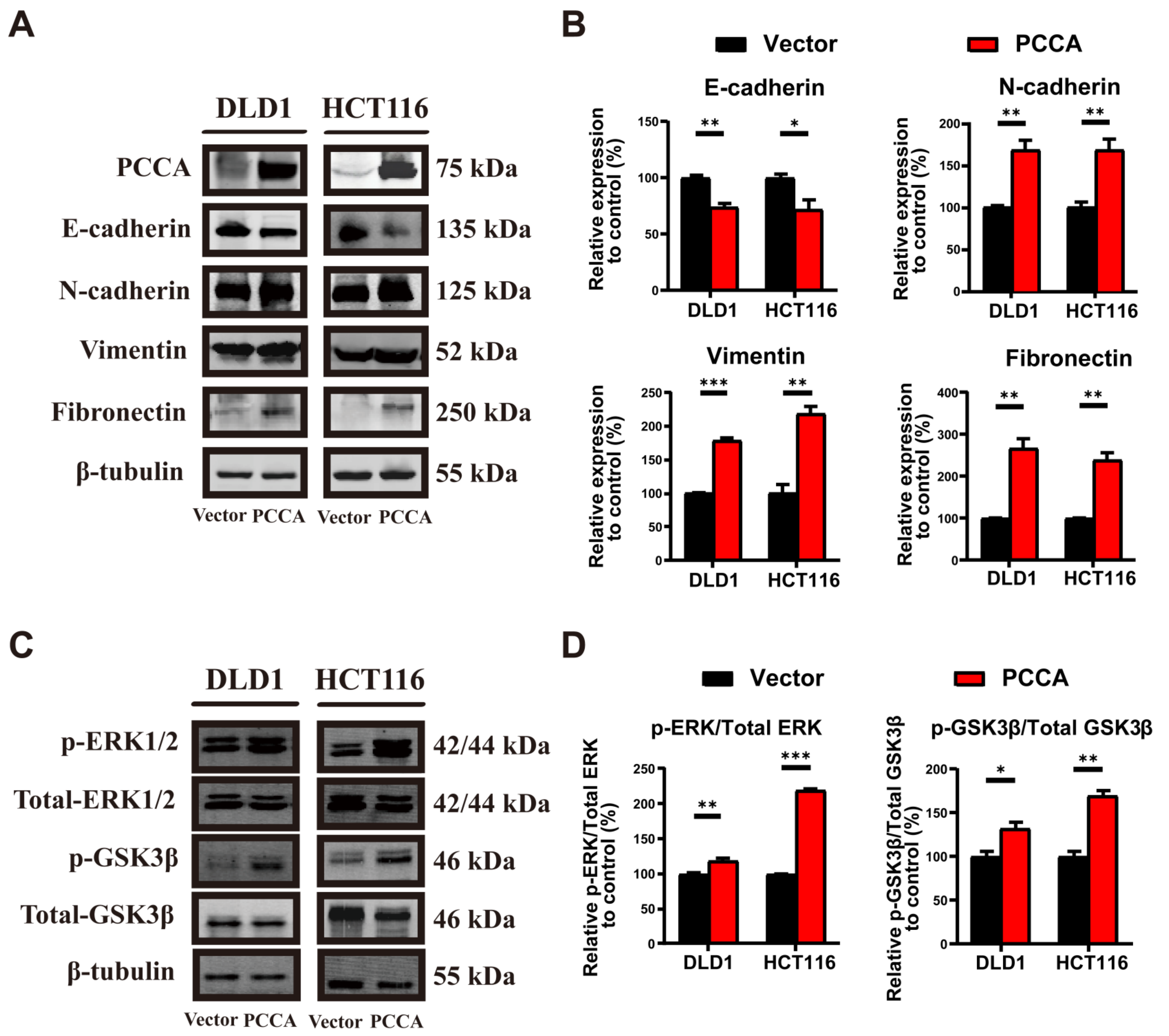

Supplementary Figure S3. Overexpression PCCA promoted tumor growth and lung metastasis of CRC

(A-B) The representative WB images and the quantification of PCCA expression in HCT116-PCCA or Vector. **(C-D)** The representative images and the quantification of colony formation in HCT116-PCCA or Vector. **(E)** The diagram of the subcutaneous tumor and lung metastasis models. **(F)** The tumor growth curves of HCT116-PCCA or Vector in nude mice (n=7). **(G-H)** The images and results of lung metastasis after 6 weeks of tumor cell tail vein injection (n=9). Data are from one experiment representative of two (**E-H**) or three (**A-D**) independent experiments with similar results. PCCA, stable transformation strain of HCT116 with *PCCA* cDNA plasmid; Vector, stable transformation strain of HCT116 with vector plasmid. The data are presented as Means ± SEM (*$P < 0.05$; ** $P < 0.01$; ***$P < 0.001$).

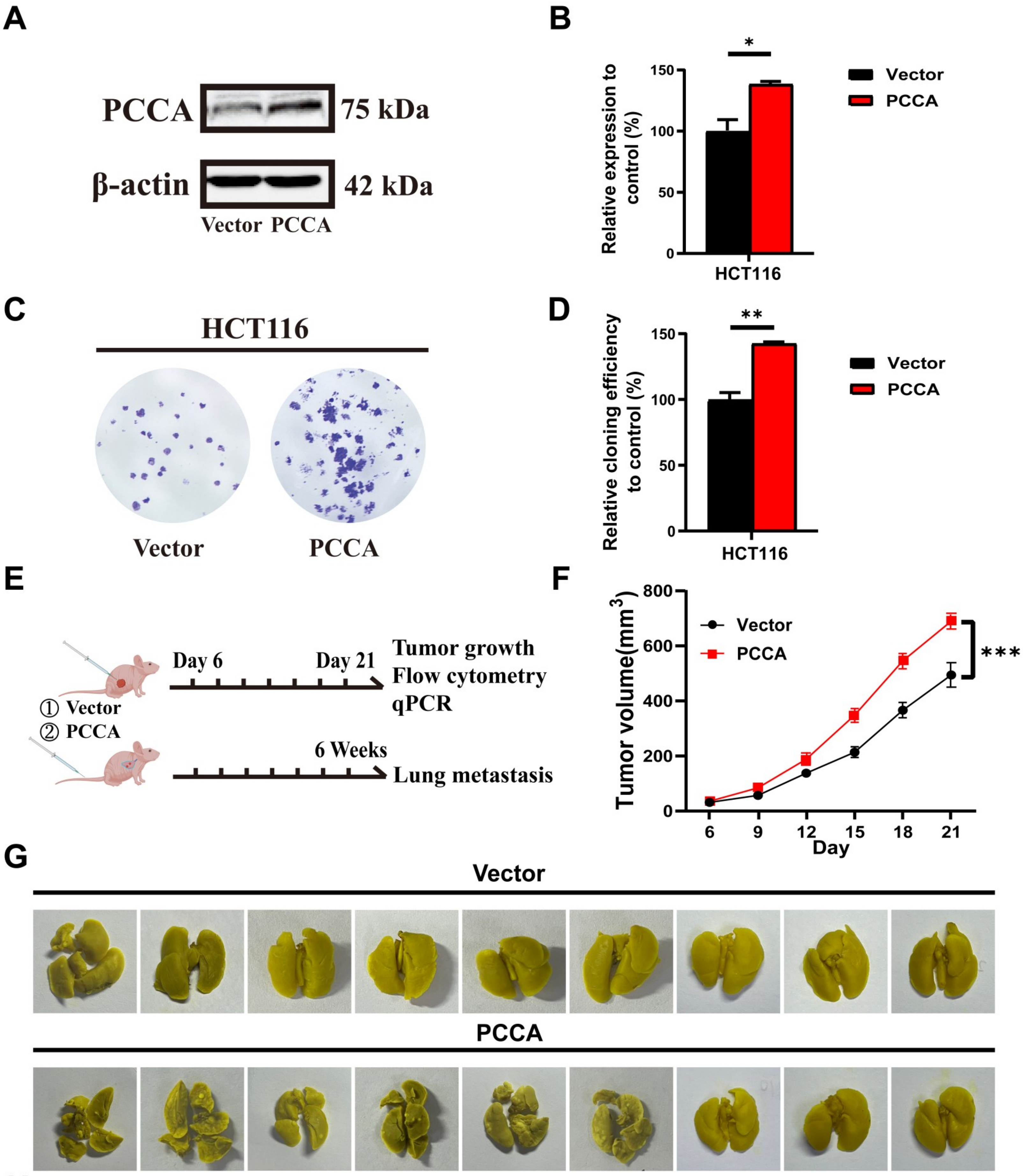

H

Group	Lung metastasis	Total	Lung metastasis/Total	*P*
Vector	1	9	1/9	0.025
PCCA	6	9	6/9	

Supplementary Figure S4. Overexpression PCCA decreased the proportions of M1-like TAMs

(A-B) The representative flow cytometry dot plots depicting the polarization of TAMs and the statistical analysis of the proportions of myeloid cells ($CD11b^{+}$) and TAMs in leukocytes ($CD45^{+}$), as well as the fold changes of M1-like ($CD11c^{+}CD206^{-}$) and M2-like ($CD11c^{-}CD206^{+}$) TAMs in the tumor tissues of HCT116-PCCA or Vector (n=7). **(C)** The heatmap showing the transcription levels of M1 and M2 marker genes in the tumor tissue of HCT116-PCCA or Vector (n=7). **(D)** The transcription levels of *Nos2* (from mice) and *NOS2* (from humans) in the tumor tissues of HCT116-PCCA or Vector (n=7). **(E)** The transcription levels of M1 and M2 marker genes in bone marrow-derived macrophages co-cultured with the conditioned media from HCT116-PCCA or Vector tumor cell cultures. The experiment was conducted in triplicates. PCCA, stable transformation strain of HCT116 with *PCCA* cDNA plasmid; Vector, stable transformation strain of HCT116 with vector plasmid. The data are presented as Means ± SEM (*$P < 0.05$; ** $P < 0.01$; ***$P < 0.001$).

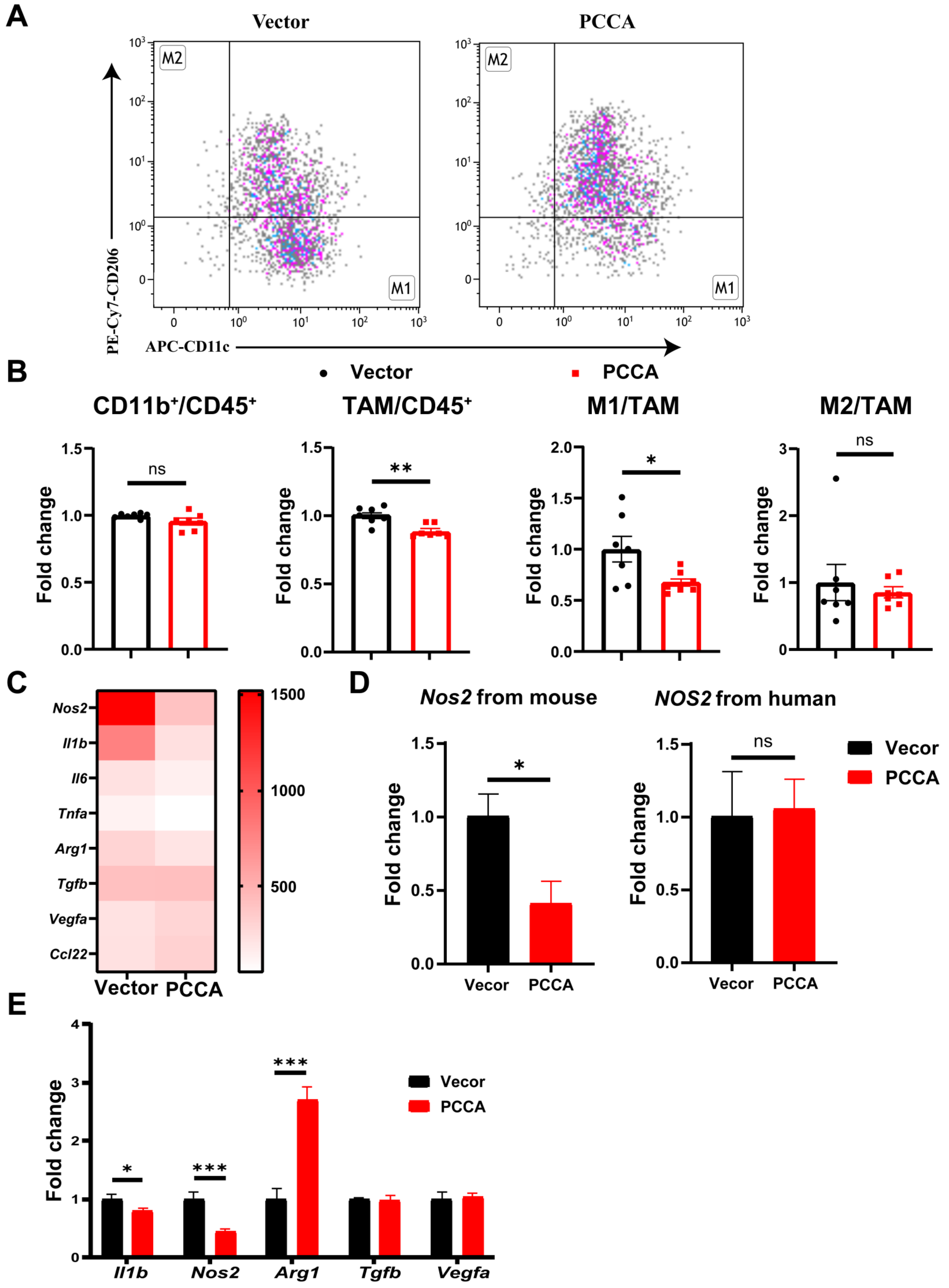

Supplement: Supplementary file 1 — Supplementary figures and tables. [file ijmsv22p0087s1.pdf]
